# Supplementary material for: Protective Effect of Avenanthramide-C on Auditory Hair Cells against Oxidative Stress, Inflammatory Cytokines, and DNA Damage in Cisplatin-Induced Ototoxicity
Source: Int J Mol Sci. 2023 Feb 2;24(3):2947. doi: 10.3390/ijms24032947 (PMC9918115; doi:10.3390/ijms24032947)
Supplement: Supplementary file 1 [file ijms-24-02947-s001.zip › ijms-2081247-supplementary.pdf]

A

1 Week

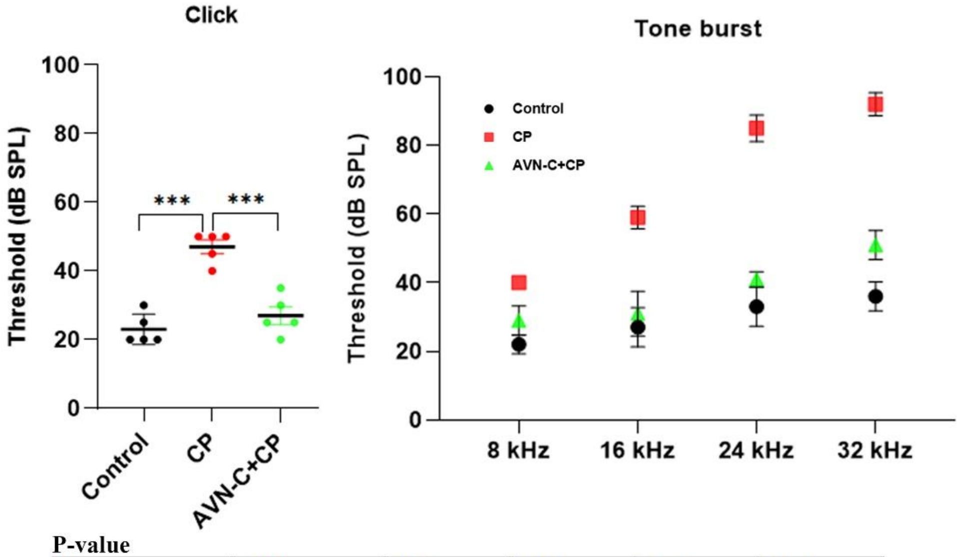

P-value

Name	*Click*	*8kHz*	*16kHz*	*24kHz*	*32kHz*
Ctl/CP	*0.0000014*	*0.0000093*	*0.0000301*	*0.0000018*	*0.0000003*
AV+CP/CP	*0.0000812*	*0.0010134*	*0.0001114*	*0.0000021*	*0.0000028*

B

2 Weeks

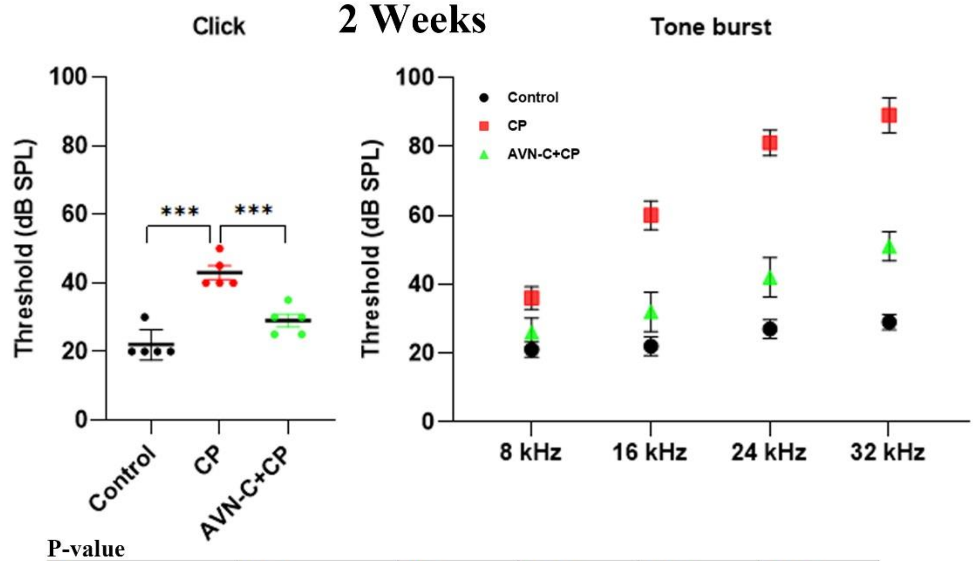

P-value

Name	*Click*	*8kHz*	*16kHz*	*24kHz*	*32kHz*
Ctl/CP	*0.000005*	*0.001256*	*0.000012*	*0.000000*	*0.000001*
AV+CP/CP	*0.000332*	*0.015180*	*0.000223*	*0.000012*	*0.000057*

Figure S1. Auditory brainstem response (ABR) by AVN-C+CP treatment. (A). ABR was performed for 1 week. (B) ABR performed at 2 weeks. (***$p \leq 0.001$, $p \leq 0.05$ was considered significant in all experimental groups).

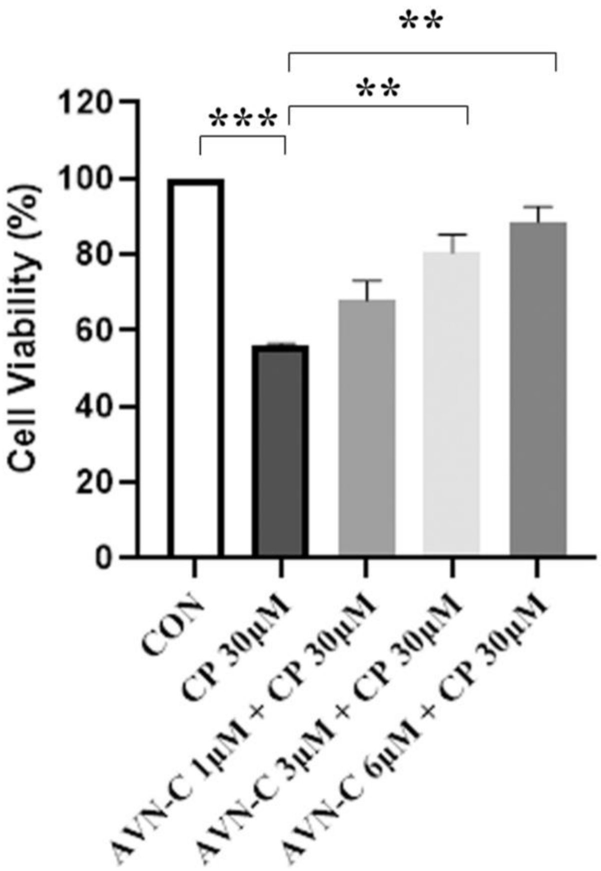

Figure S2. AVN-C Dose Cell viability assessment by MTT assay. The protection of AVN-C was examined and the level of significant protection was determined using various doses of AVN-C combined with 30 µM of CP. AVN-C 3 µM and 6 considerably protected against CP cytotoxicity on HEI-OC1 cells (** $p \leq 0.01$ AVN-C 3 µM vs. CP 30 µM; ** $p \leq 0.01$ AVN-C 6 µM vs. CP 30 µM; and *** $p \leq 0.001$ control vs. CP 30 µM) (n = 3 each). One way-ANOVA was used and $p \leq 0.05$ indicated significance.
